# Deep Learning in Different Ultrasound Methods for Breast Cancer, from Diagnosis to Prognosis: Current Trends, Challenges, and an Analysis

**DOI:** 10.3390/cancers15123139

**Published:** 2023-06-10

**Authors:** Humayra Afrin, Nicholas B. Larson, Mostafa Fatemi, Azra Alizad

**Affiliations:** 1 Department of Physiology and Biomedical Engineering, Mayo Clinic College of Medicine and Science, Rochester, MN 55905, USA; 2 Department of Quantitative Health Sciences, Mayo Clinic College of Medicine and Science, Rochester, MN 55905, USA; 3 Department of Radiology, Mayo Clinic College of Medicine and Science, Rochester, MN 55905, USA

**Keywords:** deep learning, ultrasound modalities, breast cancer, classification, segmentation, breast cancer diagnosis

## Abstract

**Simple Summary:**

Breast cancer is one of the leading causes of cancer death among women. Ultrasound is a harmless imaging modality used to help make decisions about who should undergo biopsies and several aspects of breast cancer management. It shows high false positivity due to high operator dependency and has the potential to make overall breast mass management cost-effective. Deep learning, a variant of artificial intelligence, may be very useful to reduce the workload of ultrasound operators in resource-limited settings. These deep learning models have been tested for various aspects of the diagnosis of breast masses, but there is not enough research on their impact beyond diagnosis and which methods of ultrasound have been mostly used. This article reviews current trends in research on various deep learning models for breast cancer management, including limitations and future directions for further research.

**Abstract:**

Breast cancer is the second-leading cause of mortality among women around the world. Ultrasound (US) is one of the noninvasive imaging modalities used to diagnose breast lesions and monitor the prognosis of cancer patients. It has the highest sensitivity for diagnosing breast masses, but it shows increased false negativity due to its high operator dependency. Underserved areas do not have sufficient US expertise to diagnose breast lesions, resulting in delayed management of breast lesions. Deep learning neural networks may have the potential to facilitate early decision-making by physicians by rapidly yet accurately diagnosing and monitoring their prognosis. This article reviews the recent research trends on neural networks for breast mass ultrasound, including and beyond diagnosis. We discussed original research recently conducted to analyze which modes of ultrasound and which models have been used for which purposes, and where they show the best performance. Our analysis reveals that lesion classification showed the highest performance compared to those used for other purposes. We also found that fewer studies were performed for prognosis than diagnosis. We also discussed the limitations and future directions of ongoing research on neural networks for breast ultrasound.

## 1. Introduction

Breast cancer is the leading cause of cancer worldwide and the second leading cause of death among women [1]. Ultrasound (US) is used in conjunction with mammography to screen for and diagnose breast mass, particularly in dense breasts. US has the potential to reduce the overall cost of breast cancer management as well as it can reduce benign open biopsies by facilitating fine needle aspiration, which is preferable because of its high sensitivity, specificity, and limited invasiveness [2,3,4,5]. The BI-RADS classification helps distinguish patients who need followup imaging from patients who require diagnostic biopsy [6] Moreover, intraoperative use can localize breast cancer in a cost-effective fashion and reduces the tumor-involved margin rate, eventually reducing the costs of additional management [7,8]. However, one of the major disadvantages of ultrasonography is high operator dependency, which increases the false-negative rate [9].

Thus, deep learning may come into play in reducing the manual workload of operators, creating a new role for doctors. Incorporation of deep learning models into ultrasound may have the potential to reduce the false-negative rate and reduce the overall cost of breast cancer management. It can help physicians and patients make prompt decisions by detecting, diagnosing, and monitoring the prognosis and treatment progress with considerable accuracy and time efficiency. This possibility has created considerable enthusiasm, but it also needs critical evaluation.

There have been several review papers published in the last decade on the role of deep learning in ultrasound for breast cancer segmentation and classification. They mostly combined deep learning models with B mode, shear wave elastography (SWE), color Doppler images, and sometimes with other imaging modalities [10,11,12,13,14,15]. Several surveys have been published on deep learning and machine learning models with B mode and SWE images, as well as multimodality images for breast cancer classification [16,17,18]. There are several concerns, such as bias in favor of the new model and whether the findings are generalizable and applicable to real-world settings. There are a considerable number of deep learning models developed to study breast cancer automatic segmentation and classification, but there is a lack of data on how they are improving the overall management of breast cancer, starting from screening to diagnosis and ultimately to survival. There are insufficient data on which modes of ultrasound are being used for deep learning algorithms as well.

This article reviews the current research trends on deep learning models in different ultrasound modalities for breast cancer management, from screening to diagnosis to prognosis, and the future challenges and directions of the application of these models.

## 2. Imaging Modalities Used in Breast Lesions

**Figure 1:**
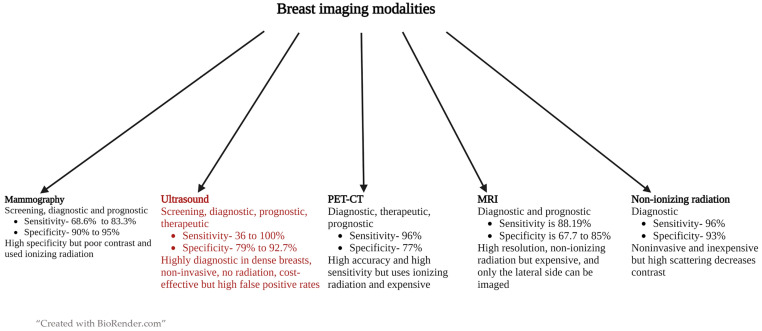
Various imaging modalities used for breast mass management, with ultrasound showing the highest sensitivity [20,21].

Various imaging modalities are used to diagnose breast masses. Self-examination, mammography, and ultrasound are usually used for screening, and if a mass is found, ultrasonography and/or MRI are usually preformed to evaluate the lesion [19]. Ultrasound has been used in various stages of breast cancer management including screening of dense breasts, diagnosis, and prognosis during chemotherapy due to its noninvasive nature, nonuse of ionizing radiation, portability, real-time nature to enable guidance for biopsies, and cost-effectiveness. Figure 1 shows different modalities of imaging that are used for breast mass management including their sensitivity, specificity, advantages, and disadvantages. Ultrasound technology, which has been advancing, includes various methods such as color Doppler, power Doppler, contrast-enhanced US, 3D ultrasound, automated breast ultrasound (ABUS), and elastography. These methods have been increasing the sensitivity and specificity of conventional US to a maximum level [20,21].

## 3. Computer-Aided Diagnosis and Machine Learning in Breast Ultrasound

Computer-aided diagnosis (CAD) can combine the use of machine learning and deep learning models and multidisciplinary knowledge to make a diagnosis of a breast mass [22]. Handheld US has been supplemented with automated breast US (ABUS) to reduce intraoperator variability [23]. The impact of 3D ABUS as a screening modality has been investigated for breast cancer detection in dense breasts as the CAD system substantially decreases interpretation time [23]. In the case of diagnosis, several studies have shown that 3D ABUS can help in the detection of breast lesions and the distinction of malignant from benign lesions [24], predicting the extent of breast lesion [25], monitoring response to neoadjuvant chemotherapy [26], and correlating with molecular subtypes of breast cancer [27], with a high interobserver agreeability [23,28]. A study proposed a computer-aided diagnosis system using a super-resolution algorithm and used a set of low-resolution images to reconstruct a high-resolution image to improve the texture analysis methods for breast tumor classification [29].

In machine learning, features are discerned and encoded by expert humans that may appear distinctive in the data and organized or segregated with statistical techniques according to these features [30,31]. Research on various machine learning models for the classification of benign and malignant breast masses has been published in the past decade [32]. Most recent papers used the k-nearest neighbors algorithm, support vector machine, multiple discriminant analysis, Probabilistic-ANN (Artificial Neural Network), logistic regression, random forest, decision tree, naïve Bayes and AdaBoost for diagnosis and classification of breast mass, binary logistic regression for classification of BI-RADS category 3a, and linear discriminate analysis (LDA) for analysis of axillary lymph node status in breast cancer patients [32,33,34,35,36,37].

## 4. What Is Deep Learning and How It Is Different

Deep learning (DL) is part of a broader family of machine learning methods that mimic the way the human brain learns. DL utilizes multiple layers to gather knowledge, and the convolution of the learned features increases in a sequential layer-wise manner [30]. Unlike machine learning, deep learning requires little to no human intervention and uses multiple layers instead of a single layer. DL algorithms have also been applied in cancer images from various modalities to make a diagnosis or classification, lesion segmentation, etc. [38]. These algorithms have been used to incorporate various clinical or histopathological data to make cancer diagnoses as well in some studies.

There are various types of convolutional neural networks. The important parts of CNNs are the input layer, output layer, convolutional layers, max-pooling layers, and fully connected layers [30,39]. The input layer should be the same as the raw or input data [30,39]. The output layer should be the same as the teaching data [30,39]. In the case of classification tasks, the unit numbers in the output layer must be the same as the category numbers in the teaching data [30,39]. The layers which are present between the input and the output layers are called hidden layers [30,39].

These multiple convolutional, fully connected, and pooling layers facilitate the learning of more features [30,39]. Usually, the convolution layer, after extracting a feature from the input image, passes to the next layer [30,39]. Convolution maintains the relationships between the pixels and results in activation [30,39]. The recurrent application of a similar filter to the input creates a map of activation, called a feature map, which facilitates revealing the intensity and location of the features recognized in the input [30,39]. The pooling layers adjust the spatial size of the activation signals to minimize the possibility of overfitting [30,39]. Spatial pooling is similar to downsampling, which adjusts the dimensionality of each map, retaining important information. Max pooling has been the commonest type of spatial pooling [30,39].

**Figure 2:**
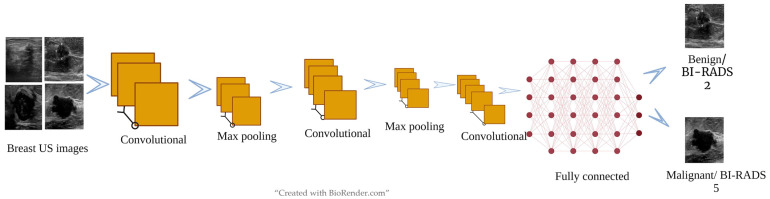
Overview of a CNN.

The function of a fully connected layer is to obtain the results from the convolutional/pooling layers and utilize them to classify the information such as images into labels [30,39]. Fully connected layers help connect all neurons in one layer to all neurons in the next layer through a linear transformation process [30,39]. The signal is then output via an activation function to the next layer of neurons [30,39]. The rectified linear unit (Relu) function is commonly used as the activation function, which is a nonlinear transformation [30,39]. The output layer is the final layer producing the given outputs [30,39]. Figure 2 shows the overview of a deep learning network.

## 5. IoT Technology in Breast Mass Diagnosis

Recently, the Industrial Internet of Things (IIoT) has emerged as one of the fastest-developing networks able to collect and exchange huge amounts of data using sensors in the medical field [40]. When it is used in the therapeutic or surgical field, it is sometimes termed the “Internet of Medical Things” (IoMT) or the “Internet of Surgical Things” (IoST), respectively [41,42,43,44]. IoMT implies a networked infrastructure of medical devices, applications, health systems, and services. It assesses the physical properties by using portable gadgets with integration into AI methods, often enabling wireless and remote devices [45,46]. This technology is improving remote patient monitoring, diagnosis of diseases, and efficient treatment via telehealth services maintained by both patients and caregivers [47]. Ragab et al. [48], developed an ensemble deep learning-based clinical decision support system for breast cancer diagnosis using ultrasound images.

Singh et al. introduced an IoT-based deep learning model to diagnose breast lesions using pathological datasets [49]. A study suggested a sensor system using temperature datasets has the potential to identify early breast mass with a wearable IoT jacket [50]. One study proposed an IoT-cloud-based health care (ICHC) system framework for breast health monitoring [51]. Peta et al. proposed an IoT-based deep max-out network to classify breast mass using a breast dataset [52]. However, most of these studies did not specify what kind of dataset they used. Image-Guided Surgery (IGS) using IoT networks may have the potential to improve surgical outcomes in surgeries where maximum precision is required in anatomical landmark tracking and instruments as well [44]. However, there is no study on IoST-based techniques involving breast US datasets.

## 6. Methods

Medline and Google Scholar databases were searched for research conducted between 2017 and February 2023 using the following terms: “deep learning models”, “breast ultrasound”, “breast lesion segmentation”, “classification”, “detection and diagnosis”, “prediction of lymph node metastasis”, “response to anticancer therapy”, “prognosis”, and “management”. After analyzing around 130 papers, we decided to exclude review papers, surveys on deep learning, and papers regarding machine learning models for breast ultrasound. We also excluded articles that didn’t specify the DL models that had been used. We finalized the list to include 59 papers focused on primary research carried out on deep learning models for breast mass ultrasound. EndNote, the reference management tool, was used to detect duplicates. The final step of the review process was to evaluate the whole manuscript to exclude articles that were deemed unnecessary.

## 7. Discussion

Various deep learning models have been tested on different stages of breast lesion management. Table 1 shown below presents all the original research conducted on breast lesion management from 2017 to February 2023, according to our search. Table 2 shows the architectures, hyperparameters, limitations, and performance metrics of the deep learning neural networks used in those studies. Most studies focused on categorizing breast lesions as benign or malignant. Five studies were performed on the BI-RADS classification of breast lesions. There is only one study on breast cyst classification. There are two studies on the distinction between benign subtypes. There are only three studies on the classification of breast carcinoma subtypes. Segmentation is the second-most common step that deep learning models were applied to. Numerous deep learning studies on segmentation may have the potential to detect tumors on screening in the future in resource-limited settings. Seven studies were conducted on the prediction of axillary lymph node metastasis. There are three studies on the prediction of response to chemotherapy. One study tested a deep learning model for segmentation during breast surgery to improve the accuracy of tumor resection and evaluate the negative margin.

Segmentation of breast mass is an important earlier step in diagnosing and characterizing mass, as is the followup on a mass once diagnosed. The most common model used in breast mass segmentation is U-Net (See Table 2). U-Net is a CNN, which is basically an encoder–decoder architecture for feature extraction and localization [53,54,55,56]. Attention U-Net is another model that was used for segmentation purposes which introduces attention layers into the U-Net to identify and focus on relevant areas such as margins or salient features of the mass to efficiently extract features [57,58]. SegNet is another encoder–decoder-based architecture that can provide semantic segmentation by using skip connections and preserving contextual information, improving margin delineation capability [59,60]. Mask R-CNN, used in another study, can provide both pixel-level segmentation and object detection [61]. Various studies used different modules other than neural networks to extract features such as transformer-based methods, local nakagami distributions, etc., and combined them to the CNN or introduced an attention layer to the CNN or modified the original CNN by adding an additional residual layer or layers to obtain an output to improve missed detections or false detections. These models can efficiently (compared to radiologists) segment the breast mass in US images within a very short amount of time.

Classifying the breast mass into benign and malignant, or BI-RADS categories, using ultrasound can help facilitate earlier decision-making in breast mass management. AlexNet [62,63,64], VGG [65,66], ResNet [62,63,65,67,68,69,70], and Inception [62,65,69], including GoogleNet, Faster R-CNN [63,66,70], and generative adversarial networks [62,71], were mostly used for breast mass classification during this period (stated in Table 1 and Table 2). AlexNet is composed of multiple convolutional layers, pooling layers, fully connected layers, and a softmax classifier [62,63,64]. VGG is composed of 16 or 19 weight layers, 3 × 3 convolutional filters, and max-pooling layers to extract features [65,66]. ResNet uses residual connections to learn residual mapping [62,63,65,67,68,69,70]. Inception architectures including GoogleNet use parallel convolutional layers of varying sizes to capture features of multiple scales [62,65,69]. Faster R-CNN is an improved model from R-CNN, which initially extracts features using a backbone CNN (such as VGG or ResNet) then predicts region of interests, the features of which are again pooled to undergo classification, bounding box regression, and finally nonmaximum suppression, improving efficacy significantly [63,66,70]. Generative adversarial network is composed of a generator and a discriminator [62,71]. The generator can map the input to generate data, which resemble the real data [62,71]. The discriminator component usually distinguishes between the real and generated data [62,71]. They are usually trained in an adversarial manner, using two separate loss functions [62,71]. These models can mimic radiologist performance in classifying breast mass into benign and malignant or BI-RADS categories in an efficient manner. Only one study predicted the molecular subtype [72].

Axillary lymph node metastasis detection is an important prognostic indicator for breast mass management, and its early detection by ultrasound can be valuable in making this whole management cost-effective and less burdensome for patients. DenseNet [73,74,75], Inception [76], ResNet [73,76,77], VGG [78], ANN [79], Xception [80], and Mask R-CNN [73] were used in the prediction of lymph node metastasis (stated in Table 2). DenseNet is composed of densely connected layer in a feed-forward manner where feature maps from all the preceding layers are concatenated in a residual manner [73,74,75]. In Artificial Neural Network (ANN), an input layer, one or more hidden layers and an output layer exist where the weights are learned independently and do not consider the relationship with neighboring data [79]. Xception is a modified version of Inception that uses depthwise separable convolutions to reduce the number of parameters to allow more efficient learning of the features [80].

Monitoring the response of the mass by ultrasound to chemotherapy can be very cost-effective for cancer patients, as it can help switch the chemotherapy regimen earlier if there is not a desirable response to the current ongoing therapy. ResNet [81] and VGG19 [82] were used in the prediction of response to chemotherapy (stated in Table 2). Most studies compared one model with another model or models or used the same model on different datasets. Around 15 studies compared these deep learning models with radiologists’ performance [65,76,78,79,80,82,83,84,85,86,87,88,89,90,91]. Mostly automatic classification and prediction of lymph node metastasis were compared with radiologists’ performance.

**Figure 3:**
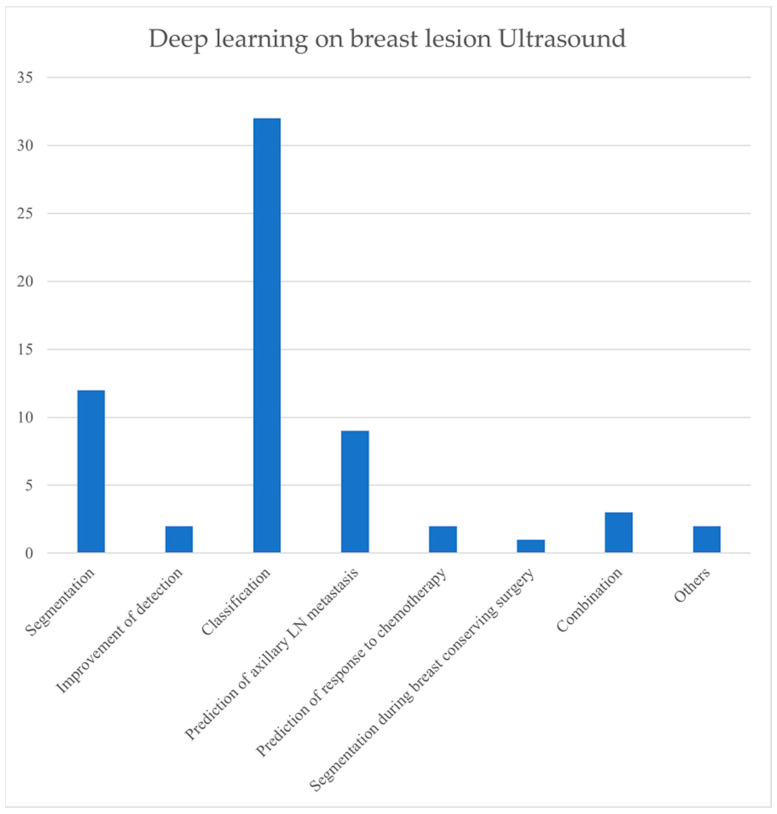
Comparison among purposes for which deep learning models are applied (No. of studies conducted).

**Figure 4:**
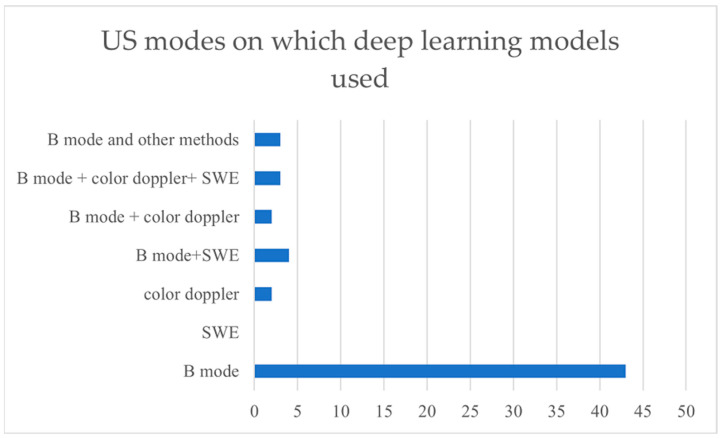
Comparison among different modes of US where deep learning models are applied (No. of studies conducted).

Over 40 studies focused only on B-mode images (See Table 1). Four studies were on B-mode and SWE combined mode. Two studies were on color Doppler mode only, and two studies were on combined B-mode and color Doppler images. Three studies were on combined B-mode, SWE, and color Doppler US images. Figure 3 shows a comparison of the purposes for which deep learning models are applied. Figure 4 shows a comparison among different modes of ultrasound where deep learning models are applied.

Adam is the most commonly used optimizer for optimizing the models in those studies (stated in Table 2), followed by stochastic gradient descent. Cross-entropy is the most used loss function. ReLU and Softmax are the most used activation functions. Image size 256 × 256 was most used as input, followed by 224 × 224 pixels and 128 × 128 pixels. The range of learning rates used in those studies is 5 × 10^−6^ to 0.01. The range of epoch numbers used in all the studies is 10–300. The range of batch sizes used in those studies is 1–128. However, the hyperparameters and parameters were not well defined in many studies. Moreover, it is difficult to understand whether fine tuning the hyperparameters can affect the performance of the models because the performance metrics used in those studies are heterogeneous.

Table 3 shows the descriptive comparative analysis across deep learning model performances among various stages of breast lesion management. This shows that deep learning models used for classification showed the best performance, with a performance metric approaching 100% [65], followed by segmentation, prediction of axillary lymph node status, and prediction of response to chemotherapy. However, the datasets, the structures of the model, and the performance metrics used by those studies were heterogeneous, so some of those metrics could not be incorporated into the analysis. Moreover, a significant number of segmentation studies used both the Dice measure and accuracy as performance metrics, so the studies overlapped between those metrics. The same phenomenon happened between accuracy and AUC, used by the classification, prediction of ALN status, and prediction of response to chemotherapy studies.

Regarding the limitations mentioned (stated in Table 2) in the studies, the most common limitation is a small dataset. However, it is difficult to define whether the dataset is adequate; most of the studies considered their datasets small or large based on the related works that had been conducted previously, whether they contained a diverse range of data or not, or by comparing the datasets with the data used in benchmark models. Using single-center samples is another commonly mentioned limitation due to its effect on making the model less generalizable. Most of the studies were retrospective, making it hard to identify if they could be applied to a real-world setting. Samples can be biased sometimes, containing more benign than malignant images or vice versa. Another limitation mentioned is that when the features of the normal region are close to the features of the mass, there is mis-segmentation. Segmentation becomes difficult when the boundary is unclear, the intensity is heterogeneous, and the features are complex. Some complex models are memory- and time-consuming, making their applicability to embedded devices very difficult. Overfitting occurs when the depth and complexity of the model cannot handle small-scale image samples. Variation exists in the results due to the involvement of more than one radiologist.

In this study, we included all the deep learning models used in different US systems for breast mass management since 2017. There are several studies on breast cancer diagnosis, but very few studies are available on axillary lymph node metastasis and the overall prognosis. A significant number of studies did not carry out any comparisons with health care professionals. Very few studies have also been conducted on multimodality US images. A considerable number of deep learning models have not been tested on the datasets. The same model has been tested on various datasets, the datasets which were collected for other reasons, making those studies retrospective [92]. Lack of standardization while extracting features can be another issue [11]. Very few prospective studies were conducted for deep learning models. Some studies confused the terminology, such as the validation set with the test set. The metrics used in the field of computer science, such as Jaccard, accuracy, precision, dice coefficient, and F1 score, were the only measures for diagnostic performance in most of the studies [93]. Most of the studies did not include datasets that have clinical information, such as age, severity, etc., which can also affect the diagnostic performance. Additionally, there is no study on how these models may improve the overall cost of breast cancer management.

Since the datasets and the models were heterogeneous, comparing the performance of each model can be quite challenging. Comparing the classifiers used and whether fine-tuning the hyperparameters affects the performance or not can be a very challenging task due to heterogeneous dataset and performance metrics. A good number of studies did not mention their limitations, which can create bias towards that model. A considerable number of studies did not mention their hyperparameters in a well-defined manner, which have the potential to affect the computational time. A significant number of studies did not mention the computational time, which can be a very essential metric to understand whether the model can be used in a real-world setting. Additionally, fewer studies were conducted for monitoring prognosis than diagnosis, so further studies are needed in those areas.

Ultrasound often misses certain types of breast mass, such as the depth of invasive micropapillary carcinoma [94,95,96], ductal carcinoma in situ, invasive lobular carcinoma, fat-surrounded isoechoic lesions, heterogeneous echoic lesions with heterogeneous backgrounds, subareolar lesions, deep lesions in huge breasts, and lesions caused by poor operator skills [97]. Delayed diagnosis and a lack of prompt management can result in lymphovascular involvement and a worse prognosis, especially in the case of rare histological breast carcinoma subtypes. A study showed micropapillary DCIS assessment using ultrasound yielded a 47% false–negative rate, and the true extent of a mass was underestimated in 81% of the cases [98]. Surgical management often requires extended surgical margins and careful preoperative axillary staging [94], which are often found by perioperative ultrasound. Some unusual histological subtypes, such as secretory breast carcinoma, show benignity on ultrasound [99] Triple hormone receptor-positive breast cancers present as isoechogenic echo textures compared to subcutaneous fat [99,100]. Triple hormone receptor-negative carcinomas, such as medullary carcinomas, appear in ultrasound as homogeneous or inhomogeneous hypoechoic masses with regular margins [99,101]. In a study of another rare type of breast cancer, metaplastic carcinoma, ultrasound was insensitive in finding primary lesions but performed better in confirming benign lesions and finding abnormal axillary lymph nodes [102]. Homogeneous hypoechoic round solid masses with posterior enhancement suggest benignity; therefore, malignant lesions showing these characteristics may show false negative results. Despite these inevitable errors, meticulous assessment of the border and internal echogenicity of the lesion can help identify its actual nature [103]. There is no study on how deep learning models could help in the detection of these rare types of breast cancer using ultrasound images, which is necessary because they show a high degree of false negativity and, therefore, missed detection, which can delay the prompt management of these patients.

Two automated breast ultrasound systems, Smart Ultrasound (Koios) for the B-mode system and QVCAD (QViewMedical), have been FDA-authorized [30]. Due to hidden layers, the basis for reaching the diagnosis cannot be shown; this is mentioned as a ‘black box problem’ in some studies, which makes it essential to develop new models that can both diagnose and show the clarity of reasoning for a dilemma [30,104].

**Table 1:** This shows original studies on deep learning models in breast mass US in various stages of breast lesion management. This table also contains the US modalities that were used, the number of images and patients, the machines and transducers used for acquisition of US images, and finally the performance metrics.

No.	Study	Year	Purpose	US Mode	No. of Images (No. of Patients)	Machine Used	Transducer	Performance Metrics
1.	Ma et al. [105]	2023	Segmentation of breast mass	B mode	780 (600), 163	Siemens ACUSON Sequoia C512 system	8.5 MHz linear	Dice coefficient: 82.46% (BUSI) and 86.78% (UDIAT)
2.	Yang et al. [106]	2023	Breast lesion segmentation	B mode	600, 780	Siemens ACUSON Sequoia C512 system, LOGIQ E9 and LOGIQ E9 Agile	8.5 MHz linear	Dice coefficient (%) 83.68 ± 1.14
3.	Cui et al. [59]	2023	Breast image segmentation	B mode	320, 647	N/A	N/A	Dice coefficient 0.9695 ± 0.0156
4.	Lyu et al. [107]	2023	Breast lesion segmentation	B mode	BUSI: 780, OASBUS: 100	N/A	N/A	Accuracy and Dice coefficient for BUSI: 97.13, and 80.71 and for OASBUD: 97.97, and 79.62 respectively.
5.	Chen et al. [60]	2023	Breast lesion segmentation	B mode	BUSI: 133 normal, 437 benign, and 210 malignant, Dataset B: 110 benign, 53 malignant	N/A	N/A	Dice coefficient 80.40 ± 2.31
6.	Yao et al. [71]	2023	Differentiation of benign and malignant breast tumors	B-mode, SWE	4580	Resona 7 ultrasound system (Mindray Medical International, Shenzhen, China), Stork diagnostic ultrasound system (Stork Healthcare Co., Ltd. Chengdu, China)	L11-3 high-frequency probe, L12-4 high-frequency probe	AUC = 0.755 (junior radiologist group), AUC = 0.781 (senior radiologist group)
7.	Jabeen et al. [108]	2022	Classification of breast mass	B mode	780 (N/A)	N/A	N/A	Accuracy: 99.1%
8.	Yan et al. [58]	2022	Breast mass segmentation	B mode	316	VINNO 70, Feino Technology Co., Ltd., Suzhou, China	5–14 MHz	Accuracy 95.81%
9.	Ashokkumar et al. [79]	2022	Predict axillary LN metastasis	B mode	1050 (850), 100 (95)	N/A	N/A	95% sensitivity, 96% specificity, and 98% accuracy
10.	Xiao et al. [109]	2022	Classification of breast tumors	B mode and tomography ultrasound imaging	120	Volusone E8 color Doppler ultrasound imaging system	The high-frequency probe was 7–12 MHz, and the volume probe frequency was 3.5–5 MHz	Specificity 82.1%, accuracy 83.8%
11.	Taleghamar et al. [81]	2022	Predict breast cancer response to neoadjuvant chemotherapy (NAC) at pretreatment	Quantitative US	(181)	RF-enabled Sonix RP system (Ultrasonix, Vancouver, BC, Canada)	L14-5/60 transducer	Accuracy of 88%, AUC curve of 0.86
12.	Ala et al. [82]	2022	Analysis of the expression and efficacy of breast hormone receptors in breast cancer patients before and after chemotherapeutic treatment	Color doppler	(120)	Color Doppler ultrasound diagnostic apparatus	LA523 probe, 4–13 MHz	Accuracy 79.7%
13.	Jiang et al. [110]	2022	Classification of breast tumors, breast cancer grading, early diagnosis of breast cancer	B mode, SWE, color doppler US	(120)	Toshiba Aplio500/400	6–13 MHz	Accuracy of breast lump detection 94.76%, differentiation into benign and malignant mass 98.22%, and breast grading 93.65%
14.	Zhao et al. [57]	2022	Breast tumor segmentation	N/A	Wisconsin Diagnostic Breast Cancer (WDBC) dataset	N/A	N/A	Dice index 0.921
15.	Althobaiti et al. [68]	2022	Breast lesion segmentation, feature extraction and classification	N/A	437 benign, 210 malignant, 133 normal	N/A	N/A	Accuracy 0.9949 (for training:test—50:50)
16.	Ozaki et al. [80]	2022	Differentiation of benign and metastatic axillary lymph nodes	B mode	300 images of normal and 328 images of breast cancer metastases	EUB-7500 scanner, Aplio XG scanner, Aplio 500 scanner	9.75-MHz linear, 8.0-MHz linear, 8.0-MHz linear	Sensitivity 94%, specificity 88%, and AUC 0.966
17.	Zhang et al. [111]	2021	Segmentation during breast conserving surgery of breast cancer patients, to improve the accuracy of tumor resection and evaluate negative margins	Color doppler US	(102)	M11 ultrasound with color Doppler	N/A	Accuracy 0.924, Jaccard 0.712
18.	Zhang et al. [112]	2021	Lesion segmentation, prediction of axillary LN metastasis	B-type, energy Doppler	(90)	E-ultrasound equipment (French acoustic department Aixplorer type)	SL15-4 probe	Accuracy 90.31%, 94.88%, 95.48%, 95.44%, and 97.65%
19.	Shen et al. [83]	2021	Reducing false-positive findings in the interpretation of breast ultrasound exams	B mode, color doppler	5,442,907	LOGIQ E9	N/A	Area under the receiver operating characteristic curve (AUROC) of 0.976
20.	Qian et al. [83]	2021	Prediction of breast malignancy risk	B-mode, colour doppler and SWE	Training set: 10,815 (634), Test set: 912 (141)	Aixplorer US system (SuperSonic Imagine)	SL15-4 or an SL10-2 linear	Bimodal AUC: 0.922, multimodal AUC: 0.955
21.	Gao et al. [66]	2021	Differentiation of benign and malignant breast nodules	B mode	(8966)	N/A	N/A	Accuracy: 0.88 ± 0.03 and 0.86 ± 0.02, respectively on two testing sets
22.	Ilesanmi et al. [55]	2021	Breast tumor segmentation	B mode	Two datasets, 264 and 830	Philips iU22, LOGIQ E9, LOGIQ E9 Agile	1–5 MHz on ML6-15-D matrix linear	Dice measure 89.73% for malignant and 89.62% for benign BUSs
23.	Wan et al. [113]	2021	Breast lesion classification	B mode	895	N/A	N/A	Random Forest accuracy: 90%, CNN accuracy: 91%, AutoML Vision (accuracy: 86%
24.	Zhang et al. [72]	2021	BI-RADS categorization of breast tumors and prediction of molecular subtype	B mode	17,226 (2542)	N/A	N/A	Accuracy, sensitivity, and specificity of 89.7, 91.3, and 86.9% for BI-RADS categorization. For the prediction of molecular subtypes, AUC of triple negative: 0.864, HER2(+): 0.811, and HR(+): 0.837
25.	Lee et al. [73]	2021	Prediction of the ALN status in patients with early-stage breast cancer	B mode	(153)	ACUSON S2000 ultrasound system (Siemens Medical Solutions, Mountain View, CA, USA)	5–14 MHz linear	Accuracy, 81.05%, sensitivity 81.36%, specificity 80.85%, and AUC 0.8054
26.	Kim et al. [65]	2021	Differential diagnosis of breast masses	B mode	1400 (971)	Philips, GE, Siemens	N/A	AUC of internal validation sets: 0.92–0.96, AUC of external validation sets: 0.86–0.90, accuracy 96–100%
27.	Zheng et al. [77]	2020	Predict axillary LN metastasis	B-mode and SWE	584 (584)	Siemens S2000 ultrasound scanner (Siemens Healthineers, Mountain View, CA, USA)	4–9 MHz linear	AUC: 0.902, accuracy of differentiation among three lymph node status: 0.805
28.	Sun et al. [74]	2020	To investigate the value of both intratumoral and peritumoral regions in ALN metastasis prediction.	B mode	2395 (479)	Hitachi Ascendus ultrasound system	13–3 MHz linear	The AUCs of CNNs in training and testing cohorts were 0.957 and 0.912 for the combined region, 0.944 and 0.775 for the peritumoral region, and 0.937 and 0.748 for the intratumoral region respectively, accuracy: 89.3%
29.	Guo et al. [75]	2020	Identification of the metastatic risk in SLN and NSLN in primary breast cancer	B mode	3049 (937)	HITACHI Vision 500 system (Hitachi Medical System, Tokyo, Japan) and Aixplorer US imaging system (SuperSonic Imagine, SSI, Aix-en-Provence, France)	linear probe of 5–13 MHz	SLNs (sensitivity = 98.4%, 95% CI 96.6–100), accuracy in test set: 74.9% and NSLNs (sensitivity = 98.4%, 95% CI 95.6–99.9), accuracy in test set: 80.2%
30.	Liang et al. [92]	2020	Classification of breast tumors	B mode	537 (221)	HITACHI Hi Vision Preirus or Ascendus, Phillips IU22, IE33, or CX50; GE Logiq E9, S6, S8, E6, or E8; Toshiba Aplio 300 or Aplio 500l, and Siemens S1000/S2000	N/A	Sensitivity 84.9%, specificity 69.0%, accuracy 75.0%, area under the curve (AUC) 0.769
31.	Chiao et al. [61]	2019	Automatic segmentation, detection, and classification of breast mass	B mode	307 (80)	LOGIQ S8, GE Medical Systems, Milwaukee, WI	9 to 12-MHz transducer	Precision 0.75, accuracy 85%
32.	Tadayyon et al. [114]	2019	Pretreatment prediction of response and 5-year recurrence-free survival of LABC patients receiving neoadjuvant chemotherapy	Quantitative US-B mode and RF data	(100)	Sonix RP system (Ultrasonix, Vancouver, Canada)	6 MHz linear array transducer (L14-5/60 W)	Accuracy 96 ± 6%, and an area under the receiver operating characteristic curve (AUC) 0.96 ± 0.08
33.	Khoshdel et al. [56]	2019	Improvement of detectability of tumors	Breast phantoms	1200 (3 phantom models)	N/A	N/A	U-Net A AUC: 0.991, U-Net B AUC: 0.975, CSI AUC: 0.894
34.	Al-Dhabyani et al. [62]	2019	Data Augmentation and classification of Breast Masses	B mode	Dataset A 780 (600), Dataset B 163	LOGIQ E9 Agile ultrasound	N/A	Accuracy 99%
35.	Zhou et al. [76]	2019	Prediction of clinically negative axillary lymph node metastasis from primary breast cancer US images.	B-mode	974 (756), 81 (78)	Philips (Amsterdam, The Netherlands; EPIQ5, EPIQ7 and IU22), Samsung (Seoul, Republic of Korea; RS80A), and GE Healthcare (Pittsburgh, PA, USA; LOGIQ E9, LOGIQ S7)	N/A	AUC of 0.89, 85% sensitivity, and 73% specificity, accuracy: 82.5%
36.	Xiao et al. [84]	2019	To increase the accuracy of classification of breast lesions with different histological types.	B mode	448 (437)	RS80A with Prestige, Samsung Medison, Co., Ltd., Seoul, Republic of Korea	3–12 MHz linear transducer	Accuracy: benign lesions: fibroadenoma 88.1%, adenosis 71.4%, intraductal papillary tumors 51.9%, inflammation 50%, and sclerosing adenosis 50%, malignant lesions: invasive ductal carcinomas 89.9%, DCIS 72.4%, and invasive lobular carcinomas 85.7%
37.	Cao et al. [63]	2019	Comparison of the performances of deep learning models for breast lesion detection and classification methods	B mode	577 benign and 464 malignant cases	LOGIQ E9 (GE) and IU-Elite (PHILIPS)	N/A	Transfer learning from the modified ImageNet produces higher accuracy than random initialization, and DenseNet provides the best result.
38.	Huang et al. [115]	2019	Classification of breast tumors into BI-RADS categories	B mode	−2238	Philips IU22 ultrasound scanner	5- to 12-MHz linear	Accuracy of 0.998 for Category “3”, 0.940 for Category “4A”, 0.734 for Category “4B”, 0.922 for Category “4C”, and 0.876 for Category “5”.
39.	Coronado-Gutierrez et al. [78]	2019	Detection of ALN metastasis from primary breast cancer	B mode	118 (105)	Acuson Antares (Siemens, Munich, Germany), MyLab 70 XVG (Esaote, Genoa, Italy)	10–13 MHz linear, 6–15 MHz linear	Accuracy 86.4%, sensitivity 84.9% and specificity 87.7%
40.	Ciritsis et al. [85]	2019	Classification of breast lesions	B mode	1019 (582)	N/A	N/A	Accuracy for BI-RADS 3–5: 87.1%, BI-RADS 2–3 vs. BI-RADS 4–5 93.1% (external 95.3%), AUC 83.8 (external 96.7)
41.	Tanaka et al. [67]	2019	Classification of breast mass	B mode	1536	N/A	N/A	Sensitivity of 90.9%, specificity of 87.0%, AUC of 0.951, accuracy of ensemble network, VGG19, and ResNet were 89%, 85.7%, and 88.3%, respectively
42.	Hijab et al. [116]	2019	breast mass classification	B mode	1300	GE Ultrasound LOGIQ E9 XDclear	Linear matrix array probe (ML6-15-D)	Accuracy 0.97, AUC 0.98
43.	Fujioka et al. [86]	2019	Distinction between benign and malignant breast tumors	B mode	Training: 947 (237), Test: 120	EUB-7500 scanner, Aplio XG scanner with a PLT-805AT	8.0-MHz linear, 8.0-MHz linear	Sensitivity of 0.958, specificity of 0.925, and accuracy of 0.925
44.	Choi et al. [87]	2019	Differentiation between benign and malignant breast masses	B mode	253 (226)	RS80A system (Samsung Medison Co., Ltd.)	3–12-MHz linear high-frequency transducer	Specificity 82.1–93.1%, accuracy 86.2–90.9%, PPV 70.4–85.2%
45.	Becker et al. [88]	2018	Classification of breast lesions	B mode	637 (632)	Logiq E9	9L linear	The training set AUC = 0.96, validation set AUC = 0.84, specificity and sensitivity were 80.4 and 84.2%, respectively
46.	Stoffel et al. [89]	2018	The distinction between phyllodes tumor (PT) and fibroadenoma (FA)	B mode	PT (36), FA (50)	Logiq E9, GE Healthcare, Chicago, IL, USA	N/A	AUC 0.73
47.	Byra et al. [90]	2018	Breast mass classification	B mode	882	Siemens Acuson (59%), GE L9 (21%), and ATL-HDI (20%)	N/A	AUC 0.890
48.	Shin et al. [70]	2018	Breast mass localization and classification	B mode	SNUBH5624 (2578), UDIAT 163	Philips (ATL HDI 5000, iU22), SuperSonic Imagine (Aixplorer), and Samsung Medison (RS80A), Siemens ACUSON Sequoia C512 system	N/A	Correct localization (CorLoc) measure 84.50%
49.	Almajalid et al. [53]	2018	Breast lesion segmentation	B mode	221	VIVID 7 (GE, Horten, Norway)	5–14 MHz linear probe	Dice coefficient 82.52%
50.	Xiao et al. [69]	2018	Breast masses discrimination	B mode	2058 (1422)	N/A	N/A	Accuracy of Transferred InceptionV3, ResNet50, transferred Xception, and CNN3 were 85.13%, 84.94%,84.06%, 74.44%, and 70.55%, respectively
51.	Qi et al. [117]	2018	Diagnosis of breast masses	B mode	8000 (2047)	Philips iU22, ATL3.HDI5000 and GE LOGIQ E9	N/A	Accuracy of Mt-Net BASIC, MIP AND REM are 93.52%, 93.89%, 94.48% and Sn-Net BASIC, MIP, and REM are 87.34%, 87.78%, 90.13%, respectively.
52.	Segni et al. [118]	2018	Classification of breast lesions	B mode, SWE	68 (61)	UGEO RS80A machinery	3–16 MHz or 3–12 MHz linear	Sensitivity > 90%, specificity 70.8%, ROC 0.81
53.	Zhou et al. [119]	2018	Breast tumor classification	B mode, SWE	540 (205)	Supersonic Aixplorer system	9–12 MHz linear	Accuracy 95.8%, sensitivity 96.2%, and specificity 95.7%
54	Kumar et al. [54]	2018	Segmentation of breast mass	B mode	433 (258)	LOGIQ E9 (General Electric; Boston, MA, USA) and IU22 (Philips; Amsterdam, The Netherlands)	N/A	Dice coefficient 84%
55.	Cho et al. [91]	2017	to improve the specificity, PPV, and accuracy of breast US	B mode, SWE and color doppler	126 (123)	Prestige; Samsung Medison, Co, Ltd.	3–12-MHz linear	Specificity 90.8%, positive predictive value PPV 86.7%, accuracy 82.4, AUC 0.815
56.	Han et al. [120]	2017	Classification of breast tumors	B mode	7408 (5151)	iU22 system (Philips, Inc.), RS80A (Samsung Medison, Inc.)	N/A	Accuracy 0.9, sensitivity 0.86, specificity 0.96.
57.	Kim et al. [121]	2017	Diagnosis of breast masses	B mode	192 (175)	RS80A with Prestige, Samsung Medison, Co. Ltd., Seoul, Republic of Korea	3–12-MHz linear	Accuracy 70.8%
58.	Yap et al. [64]	2017	Detection of breast lesions	B mode	Dataset A: 306, Dataset B: 163	B&K Medical Panther 2002 and B&K Medical Hawk 2102 US systems, Siemens ACUSON Sequoia C512 system	8–12 MHz linear,17L5 HD linear (8.5 MHz)	Transfer Learning FCN-AlexNet performed best, True Positive Fraction 0.98 for dataset A, 0.92 for dataset B
59.	Antropova et al. [122]	2017	Characterization of breast lesions	N/A	(1125)	Philips HDI5000 scanner	N/A	AUC = 0.90

**Table 2:** This table shows deep learning models used in the studies stated in Table 1 (2017–February 2023), hyperparameters, loss function, activation function, limitations, and performance metrics.

No.	Study	Purpose	Deep Learning Models	Hyperparameters	Loss Function	Activation Function	Limitations	Performance Metrics
1.	Ma et al. [105]	Segmentation of breast mass	ATFE-Net	Weights of ResNet-34, 80 epochs, batch size 8, the weight decay and momentum are set to 10^−8^ and 0.9, respectively. The initial learning rate is 0.0001. Adam optimizer is adopted, Image input size = 256 × 256 pixels	Binary cross-entropy and Dice (hybrid)	Softmax and Rectified Linear Units (ReLUs)	1. When the pixel intensity of the target region is close to mass, there is missegmentation 2. Results not relevant to classification 3. Relies on adequate manually labeled data, which are scarce	Dice coefficient: 82.46% (BUSI) and 86.78% (UDIAT)
2.	Yang et al. [106]	Breast lesion segmentation	CSwin-PNet	Swin Transformer, channel attention mechanism, gating mechanism, boundary detection (BD) module was used, the learning rate 0.0001, batch size 4 and maximum epoch number 200, image input size 224 × 224, adam optimizer, GEUL, ReLU and sigmoid activation function	Hybrid loss (Binary cross-entropy and Dice)	ReLU and sigmoid activation function	Fails to segment accurately when the lesion margin is not clear, and the intensity of the region is heterogenous.	Dice coefficient (%) 83.68 ± 1.14
3.	Cui et al. [59]	Breast image segmentation	SegNet with the LNDF ACM	MiniBatch Size 32, Initial Learn Rate 0.001, Max Epochs 50, Validation Frequency 20, image input size 128 × 128	Not specified	ReLU and Softmax	Large-scale US dataset unavailability makes it difficult to predict boundaries of blurred area accurately, loss of spatial information during downsampling	Dice coefficient 0.9695 ± 0.0156
4.	Lyu et al. [107]	Breast lesion segmentation	Pyramid Attention Network combining Attention mechanism and Multi-Scale features (AMS-PAN)	Image input size = 256 × 256 pixels, the optimizers include the first-order momentum-based SGD iterator, the second-order momentum-based RMSprop iterator, and the Adam iterator, Epoch 50 Learning rate 0.01, Batch 16, Gradient decay policy: ReduceLROnPlateau, Patience epoch 3 Decay factor 0.2	Not specified	ReLU activation function	The segmentation results are different from the ground truth in some cases, more time consuming compared to other models.	Accuracy and Dice coefficient for BUSI: 97.13, and 80.71 and for OASBUD: 97.97, and 79.62 respectively.
5.	Chen et al. [60]	Breast lesion segmentation	SegNet with deep supervision module, missed detection residual network and false detection residual network	Epoch size 50, batch size 12, initial learning rate 0.001, Optimizer: Adam optimizer	Binary-cross entropy (BCE) and mean square error (MSE)	Activation function: sigmoid activation and linear activation layers	Missed detection, false detection in individual images, more computational cost	Dice coefficient 80.40 ± 2.31
6.	Yao et al. [71]	Differentiation of benign and malignant breast tumors	Generative adversarial network	The max training epoch is 200, batch size of 1, Optimizer: Adam optimizer, learning rate 2 × 10^−4^, convolution kernels 4 × 4, Image input size = 256 × 256	MAE and Cross entropy	a Tanh activation layer, a Leaky-ReLU activation layer	Limitation of imaging hardware, due to limited cost and size. Portable US scanner’s function is limited in resource-limited settings	AUC = 0.755 (junior radiologist group), AUC = 0.781 (senior radiologist group)
7.	Jabeen et al. [108]	Classification of breast mass	DarkNet53	Learning rate 0.001, mini batch size 16, epochs 200, the learning method is the stochastic gradient descent, optimization method is Adam, reformed deferential evolution (RDE) and reformed gray wolf (RGW) optimization algorithms; image input size 256-by-256	Multiclass cross entropy loss	Sigmoid activation	The computational time is 13.599 (s), limitations not specified	Accuracy: 99.1%
8.	Yan et al. [58]	Breast mass segmentation	Attention Enhanced U-net with hybrid dilated convolution (AE U-net with HDC)	Due to the limitation of the GPU, HDC was unable to replace all upsampling and pooling operations	AE U-Net model is composed of a contraction path on the left, an expansion path on the right, and four AGS in the middle, batch size 5, epoch 60, Training_Decay was 1 × 10^−8^, initial learning rate 1 × 10^−4^, input image size 500 × 400 pixels	Binary cross-entropy	ReLU and Sigmoid	Accuracy 95.81%
9.	Ashokkumar et al. [79]	Predict axillary LN metastasis from primary breast cancer features	ANN based on feed forward, radial basis function, and Kohonen self-organizing	Batch size 32, optimizer: Adam, primary learning rate 0.0002, image input size 250 by 350 pixels,	Not specified	Not specified	Limitation not specified	95% sensitivity, 96% specificity, and 98% accuracy
10.	Xiao et al. [109]	Classification of breast tumors	Deep Neural Network model	Batch size 24	Cross entropy	Linear regression and sigmoid activation	1. Small sample size, 2. The clinical trial was conducted in a single region or a small area of multicenter, large-sample hospitals, 3. compared to light scattering imaging, sensitivity not statistically significant.	Specificity 82.1%, accuracy 83.8%
11.	Taleghamar et al. [81]	Predict breast cancer response to neo-adjuvant chemotherapy (NAC) at pretreatment	ResNet, RAN56	Image input size 512 × 512 pixel, learning rate = 0.0001, dropout rate = 0.5, cost weight = 5, batch size = 8, Adam optimizer was used,	Cross entropy	ReLU	Relatively small dataset, resulting in overfitting and lack of generalizability	Accuracy of 88%, AUC curve of 0.86
12.	Ala et al. [82]	Analysis of the expression and efficacy of breast hormone receptors in breast cancer patients before and after chemotherapeutic treatment	the VGG19FCN algorithm	Not specified	Not specified	Not specified	Sample not enough, in the follow-up, the sample number needs to be expanded to further assess different indicators	Accuracy 79.7%
13.	Jiang et al. [110]	Classification of breast tumors, breast cancer grading, early diagnosis of breast cancer	Residual block and Google’s Inception module	The optimization algorithm: Adam, the maximum number of iterations: 10,000 for detection, 6000 for classification, the initial learning rate: 0.0001, weight randomly initialized, and bias initialized to 0, batch size 8	Multiclass cross entropy	Softmax	Small sample size, so the results can be biased, patient sample should be expanded in follow up studies, multicenter and large-scale study should be conducted.	accuracy of breast lump detection 94.76%, differentiation into benign and malignant mass 98.22%, and breast grading 93.65%
14.	Zhao et al. [57]	Breast tumor segmentation	U-Net and attention mechanism	Learning rate = 0.00015, Adam optimizer was used	Binary cross entropy (BCE), Dice loss, combination of both	ReLU	Only studies the shape feature constraints of masses	Dice index 0.921
15.	Althobaiti et al. [68]	Breast lesion segmentation, feature extraction and classification	LEDNet, ResNet-18, Optimal RNN, SEO	Not specified	Not specified	Softmax	Not specified	Accuracy 0.9949 (for training:test—50:50)
16.	Ozaki et al. [80]	Differentiation of benign and metastatic axillary lymph nodes	Xception	Image input size: 128 × 128-pixel, optimizer algorithm = Adam, Epoch: 100,	Categorical cross entropy	Softmax	1. The study was held at a single hospital, collecting images at multiple institutions are needed. 2. training and test data randomly contained US images with different focus, gain, and scale, affecting the training and subsequently diagnostic performance of the DL. 3. trimming process may have lost some information, influencing the performance of the model. 4. some of the ultrasound images can be overlapped. The model might have remembered same images or have diagnosed on the basis of surrounding tissues, rather than on the lymph node itself.	Sensitivity 94%, specificity 88%, and AUC 0.966
17.	Zhang et al. [111]	Segmentation during breast conserving surgery of breast cancer patients, to improve the AC of tumor resection and negative margins	Deep LDL model	Not specified	Cross entropy	Softmax	Small number of patients, not generalizable to all tumors, especially complicated tumor edge characteristics	Accuracy 0.924, Jaccard 0.712
18.	Zhang et al. [112]	Lesion segmentation, prediction of axillary LN metastasis	Back propagation neural network	Not specified	Not specified	Not specified	Study samples were small, lacks comparison with DL algorithms, low representativeness	Accuracy 90.31%, 94.88%, 95.48%, 95.44%, and 97.65%
19.	Shen et al. [83]	Reducing false-positive findings in the interpretation of breast ultrasound exams	ResNet-18	Optimizer: Adam, epoch: 50, image input size 256 × 256 pixels, learning rate η ∈ 10[−5.5, −4], weight decay λ ∈ 10[−6, −3.5] on a logarithmic scale,	Binary cross-entropy	Sigmoid nonlinearity	1. Not multimodal imaging, 2. did not provide assessment on patient cohorts stratified by various other risk factors such as family history of breast cancer and BRCA status.	area under the receiver operating characteristic curve (AUROC) of 0.976
20.	Qian et al. [83]	Prediction of breast malignancy risk	ResNet-18 combined with the SENet backbone	Batch size 20, initial learning rate 0.0001, 50 epochs, a decay factor of 0.5, maximum iteration 13,000 steps, ADAM optimizer was used, image size 300 × 300	Cross entropy	Softmax and ReLU	1. Can only be applied to Asian populations, 2. excluded variable images from US systems other than Aixplorer, 3. not representative of the natural distribution of cancer patients, dataset only included biopsy-confirmed lesions, not those who underwent followup procedures, 4. did not include patients’ medical histories, 5. intersubject variability of US scanning such as TGC, dynamic range compression, artifacts, etc.	Bimodal AUC: 0.922, multimodal AUC: 0.955
21.	Gao et al. [66]	Classification of benign and malignant breast nodules	Faster R-CNN and VGG16, SSL	Faster R-CNN: Learning rate (0.01, 0.001, 0.0005), batch size (16, 64, 128), and L2 decay (0.001, 0.0005, 0.000), optimizer: gradient descent, iterations: 70,000, image input size: 128 × 128 pixels, gradient descent optimizer was used, momentum 0.9, iterations 70,000, SL-1 and SL-2: Learning rate (0.005, 0.003, 0.001), batch size (64.0, 128.0), iteration number (40,000.0, 100,000.0), ramp-up length (5000.0, 25,000.0, 40,000.0), ramp-down length (5000.0, 25,000.0, 40,000.0), the smoothing coefficient was 0.99, dropout probability 0.5, optimizer: Adam	Cross entropy	ReLU	Not specified	Accuracy: 0.88 ± 0.03 and 0.86 ± 0.02, respectively on two testing sets
22.	Ilesanmi et al. [55]	Breast tumor segmentation	VEU-Net	Adam optimizer, the learning rate 0.0001, 96 epochs, batch size 6, iterations 144, image input size 256 × 256 pixels	Binary cross-entropy	ReLU and sigmoid	Not specified	Dice measure 89.73% for malignant and 89.62% for benign BUSs
23.	Wan et al. [113]	Breast lesion classification	Traditional machine learning algorithms, convolutional neural network and AutoML	Input image size: 288 × 288	Binary cross entropy	Rectified Linear Units (ReLUs)	1. Images were not in DICOM format, so patient data were not available. 2. small sample size, so could not assess different classifiers in handling huge data, 3. no image preprocessing, relatively simple model, 4. relationship between image information and performance of different models are to be investigated.	Random Forest accuracy: 90%, CNN accuracy: 91%, AutoML Vision (accuracy: 86%
24.	Zhang et al. [72]	BI-RADS classification of breast tumors and prediction of molecular subtype	Xception	Cannot be accessed	Not specified	Not specified	1. Training set came from the same hospital and did not summarize information on patients and tumors, 2. small sample size, 3. retrospective, all patients undergone surgery, although some women choose observation.	Accuracy, sensitivity, and specificity of 89.7, 91.3, and 86.9% for BI-RADS categorization. For the prediction of molecular subtypes, AUC of triple negative: 0.864, HER2(+): 0.811, and HR(+): 0.837
25.	Lee et al. [73]	Prediction of the ALN status in patients with early-stage breast cancer	Mask R–CNN, DenseNet-121	Mask R-CNN: Backbone: ResNet-101, scales of RPN anchor: (16, 32, 64, 128, 256), optimizer: SGD, initial learning rate: 10−3, momentum: 0.9, weight decay: 0.01, epoch: 180, batch size: 3; DenseNet-121: optimizer: Adam, initial learning rate: 2 × 10^−5^, momentum: 0.9, epoch: 150, batch size: 16	Binary cross-entropy	Not specified	1. Small dataset, 2. More handcrafted features are to be analyzed to increase the prediction ability	Accuracy, 81.05%, sensitivity 81.36%, specificity 80.85%, and AUC 0.8054
26.	Kim et al. [65]	Differential diagnosis of breast masses	U-Net, VGG16, ResNet34, and GoogLeNet (weakly supervised)	L2 regularization, batch size 64, optimizer: Adam, with learning rate 0.001, image input size 224 × 224 pixels, a class activation map is generated using a global average pooling layer.	Not specified	Softmax	1. Not trained with a large dataset, 2. time- and labor-efficiency not directly assessed because of the complexity of data organizing process.	AUC of internal validation sets: 0.92–0.96, AUC of external validation sets: 0.86–0.90, accuracy 96–100%
27.	Zheng et al. [77]	Predict axillary LN metastasis	Resnet	Learning rate to 1 × 10^4^, Adam optimizer, Batch size 32, Maximum iteration step 5000, SVM as the classifier, image input size 224 × 224 pixels	Cross-entropy	Not specified	1. Single-center study, 2. Multifocal and bilateral breast lesions are excluded, because of difficulty in determining ALN metastatic potential, so only the potential of patients with a single lesion can be predicted, 3. Patients cannot be stratified based on their BRCA status.	AUC: 0.902, accuracy of differentiation among three lymph node status: 0.805
28.	Sun et al. [74]	To investigate the value of both intratumoral and peritumoral regions in ALN metastasis prediction.	DenseNet	Adam optimizer, a learning rate of 0.0001, batch size 16, and regularization weight 0.0001	Cross-entropy	ReLU	1. Change of depth of mass leads to misinterpretation of lesion detection, 2. Did not preprocess the image.	The AUCs of CNNs in training and testing cohorts were 0.957 and 0.912 for the combined region, 0.944 and 0.775 for the peritumoral region, and 0.937 and 0.748 for the intratumoral region respectively, accuracy: 89.3%
29.	Guo et al. [75]	Identification of the metastatic risk in SLN and NSLN (axillary) in primary breast cancer	DenseNet	Input image size 224× 224, optimizer: Adadelta algorithm, learning rate (1 × 10^−5^), 30 epochs	Cross-entropy	ReLU	1. Retrospective, 2. A limited number of hospitals, 3. Patients with incomplete data were excluded, leading to bias, 3. Not multimodal, 4. analyzed a single image at a time, could not capture the correlation between images, 5. lacks a small number of masses which is not seen in US methods.	SLNs (sensitivity = 98.4%, 95% CI 96.6–100), accuracy in test set: 74.9% and NSLNs (sensitivity = 98.4%, 95% CI 95.6–99.9), accuracy in test set: 80.2%
30.	Liang et al. [92]	Classification of breast tumors	GooLeNet and CaffeNet	Base learning rate 0.001, epoch 200, image input size 315 × 315 pixels	Not specified	Not specified	1. More parameter and data adjustments are needed, 2. Not a large sample size, not multicenter 3. Manually drawing the outline should be drawn by senior physicians which was often not possible, 4. lacks comparison with other models.	Sensitivity 84.9%, specificity 69.0%, accuracy 75.0%, area under the curve (AUC) 0.769
31.	Chiao et al. [61]	Automatic segmentation, detection and classification of breast mass	Mask R-CNN	Used region proposal network (RPN) to extract features, and to classify, mini-batch size 2, a balancing parameter of 10	Binary cross-entropy loss	Not specified	Not specified	Precision 0.75, accuracy 85%
32.	Tadayyon et al. [114]	Pre-treatment prediction of response and 5-year recurrence-free survival of LABC patients receiving neoadjuvant chemotherapy	Artificial neural network (ANN)	Single hidden layer model	Not specified	Not specified	Not specified	Accuracy 96 ± 6%, and an area under the receiver operating characteristic curve (AUC) 0.96 ± 0.08
33.	Khoshdel et al. [56]	Improvement of detectability of tumors	U-Net	Weights were initialized by Gaussian random distribution using Xavier’s method, batch size 10, 75 epochs, image input size 256 × 256 pixels	Not specified	Not specified	When certain breast model type is missing, AUC decreases, wide-diversity of breast types are needed.	U-Net A AUC: 0.991, U-Net B AUC: 0.975, CSI AUC: 0.894
34.	Al-Dhabyani et al. [62]	Data Augmentation and classification of Breast Masses	CNN (AlexNet) and TL (VGG16, ResNet, Inception, and NASNet), Generative Adversarial Networks	AlexNet: Adam optimizer, the learning rate 0.0001, 60 epochs, dropout rate 0.30, Transfer learning: Adam optimizer, the learning rate 0.001, epochs 10	Multinomial logistic loss	Leaky ReLU and softmax	1. Time-consuming training process and needs high computer resources, 2. Not enough real images have been collected, 3. Cannot synthesize high-resolution images using a generative model	Accuracy 99%
35.	Zhou et al. [76]	Prediction of clinically negative axillary lymph node metastasis from primary breast cancer US images.	Inception V3, Inception-ResNet V2, and ResNet-101	Adam optimizer, batch size 32, end-to-end supervised learning, initial learning rate 0.0001 and decayed by a factor of 10, epoch 300, dropout probability 0.5, augmented image size 200 × 300 pixels	Not specified	Not specified	1. Retrospective and limited size data, 2. Variations in the quality of images due to examinations being performed by multiple physicians, 3. The accuracy of LN metastasis status is dependent on the time of breast surgery, some of the patients with negative LN, if followed up for a long time, may progress to positive LNs	AUC of 0.89, 85% sensitivity, and 73% specificity, accuracy:82.5%
36.	Xiao et al. [84]	To increase the accuracy of classification of breast lesions with different histological types.	S-Detect	Not specified	Not specified	Not specified	1. Not enough cases of some rare types of breast lesions, diagnostic accuracy in these rare types needs further analyses, 2. The quality of images is better since they are obtained by an experienced radiologist, but the diagnostic performance of the DL model needs further verification.	Accuracy: benign lesions: fibroadenoma 88.1%, adenosis 71.4%, intraductal papillary tumors 51.9%, inflammation 50%, and sclerosing adenosis 50%, malignant lesions: invasive ductal carcinomas 89.9%, DCIS 72.4%, and invasive lobular carcinomas 85.7%
37.	Cao et al. [63]	Comparison of the performances of deep learning models for breast lesion detection and classification methods	AlexNet, ZFNet, VGG, ResNet, GoogLeNet, DenseNet, Fast Region-based convolutional neural networks (R-CNN), Faster R-CNN, Spatial Pyramid Pooling Net, You Only Look Once (YOLO), YOLO version 3 (YOLOv3), and Single Shot MultiBox Detector (SSD)	Image input size was different which was resized to 256 × 256 pixels, epoch: 2000	Not specified	Softmax, bounding-box regression	1. SSD300 + ZFNet is better than SSD300 + VGG16 under the benign, but worse under the malignant lesions, due to model complexity, 2. VGG16 reaches overfitting for benign lesions, 3. AlexNet, ZFNet, and VGG16 perform poorly for full images and LROI, while learning from scratch, due to the dimensionality problem, leading to over-fitting.	Transfer learning from the modified ImageNet produces higher accuracy than random initialization, and DenseNet provides the best result.
38.	Huang et al. [115]	Classification of breast tumors into BI-RADS categories	ROI-CNN, G-CNN	The minibatch size: 16 images, Optimizer: SGD (stochastic gradient descent), a learning rate of 0.0001, a momentum of 0.9, input image size 288 × 288	Dice loss, multi-class cross entropy	ReLU, softmax	Not specified	Accuracy of 0.998 for Category “3”, 0.940 for Category “4A”, 0.734 for Category “4B”, 0.922 for Category “4C”, and 0.876 for Category “5”.
39.	Coronado-Gutierrez et al. [78]	Detection of ALN metastasis from primary breast cancer	VGG-M	A variation of Fisher Vector (FV) was used for feature extraction and sparse partial least squares (PLS) were used for classification.	Not specified	Not specified	1. Because of ambiguity in diagnosis, many interesting lymph node images had to be discarded, 2. did not measure the intra-operator variability, 3. small size of the dataset, was needed to confirm these results in a large multicenter area.	Accuracy 86.4%, sensitivity 84.9% and specificity 87.7%
40.	Ciritsis et al. [85]	Classification of breast lesions	Deep CNN	Epoch 51, input image size: 301 × 301 pixels	Not specified	Softmax	1. Final decision depends on more information than image data, such as family history, age, and comorbidities, decided by radiologists in a clinical setting, which were not possible in this study, 2. relatively small data	Accuracy for BI-RADS 3–5: 87.1%, BI-RADS 2–3 vs. BI-RADS 4–5 93.1% (external 95.3%), AUC 83.8 (external 96.7)
41.	Tanaka et al. [67]	Classification of breast mass	VGG19, ResNet152, an ensemble network	The learning rate 0.00001 and weight decay 0.0005, epoch 50, input image size 224 × 224 pixels, batch size 64, optimizer: adaptive moment estimation (Adam), dropout 0.5	Not specified	Not specified	1. Test set was very small, 2. They targeted only women with masses found in second look, so malignant masses were there than benign ones, so this model cannot be applied to women with initial screening, 3. mass was evaluated only by one doctor, all test patches were not used for calculation.	Sensitivity of 90.9%, specificity of 87.0%, AUC of 0.951, accuracy of ensemble network, VGG19, and ResNet were 89%, 85.7%, and 88.3%, respectively
42.	Hijab et al. [116]	breast mass classification	VGG16 CNN	Optimizer: stochastic gradient descent (SGD), 50 epochs, batch size 20, learning rate 0.001	Not specified	ReLU	1. Dataset relatively small, 2. lack of demographic variety in race and ethnicity in the training data can impact the detection and survival outcomes negatively for underrepresented patient population.	Accuracy 0.97, AUC 0.98
43.	Fujioka et al. [86]	Distinction between benign and malignant breast tumors	GoogLeNet	Batch size 32, 50 epochs, image input size 256 × 256 pixels	Not specified	Not specified	1. Retrospective study at a single institution, so more extensive, multicenter studies are needed to validate the findings, 2. recurrent lesions were diagnosed using histopathology or cytology, 3. Image processing resulted in a loss of information, influencing the performance, 4. there can be an issue in adaptability of learning outcome in testing because of using other US systems.	Sensitivity of 0.958, specificity of 0.925, and accuracy of 0.925
44.	Choi et al. [87]	Differentiation between benign and malignant breast masses	GoogLeNet CNN (S-DetectTM for Breast)	Not specified	Not specified	Not specified	1. Interobserver variability may be seen in CAD results due to variation in the observed features among the representative images, 2. Not applicable to diagnosis of non-mass lesions (e.g., calcifications, architectural distortion) because they were excluded from analysis due to having not clearly distinguishable margin, 3. They included benign or potentially benign masses that were not biopsied, which were stable or diminished in size during follow-up.	Specificity 82.1–93.1%, accuracy 86.2–90.9%, PPV 70.4–85.2%
45.	Becker et al. [88]	Classification of breast lesions	Deep neural network	Not specified	Not specified	Not specified	1. Large portion of patients was excluded due to strict inclusion criteria, resulting in possibility of falsely low or high performance, 2. single-center study and large portion of benign lesions were scars, may be misdiagnosed as cancerous, 3. Retrospective, inherent selection bias, a high proportion of referred patients had a previous history of cancer or surgery, 4. small sample size.	The training set AUC = 0.96, validation set AUC = 0.84, specificity and sensitivity were 80.4 and 84.2%, respectively
46.	Stoffel et al. [89]	The distinction between phyllodes tumor and fibroadenoma from breast ultrasound images	Deep networks in ViDi Suite	Not specified	Not specified	Not specified	1. They only trained to distinguish between PT and FA, so it cannot diagnose other lesions, such as scars or invasive cancers, 2. it would accurately identify unaffected patients, rather than patients requiring treatment, 3. small sample size, 4. retrospective design in a stringent experimental setting, 5. since high prevalence of PT were in the training cohort, despite the fact FA is more common, it has potential to overestimate the occurrence of PT, 6. The cost-effectiveness of this method application has not yet been addressed.	AUC 0.73
47.	Byra et al. [90]	Breast mass classification	VGG19	The learning rate was initially 0.001 and was decreased by 0.00001 per epoch up to 0.00001. The momentum was 0.9, the batch size was 40, optimizer: stochastic gradient descent, epoch 16, dropout 80%	Binary cross-entropy	Sigmoid and ReLU	Radiologist has to identify the mass and select the region of interest	AUC 0.890
48.	Shin et al. [70]	Breast mass localization and classification	Faster R-CNN, VGG-16 net, ResNet-34, ResNet-50, and ResNet-101	Optimizer: SGD, Adam optimizer, learning rate 0.0005, weight decay of 0.0005, batch size 1 and 2,	Classification (cross entropy) and regression losses	Not specified	1. Failed to train a mass detector due to poor image quality, unclear boundary, insufficient, confused and complex features, such as irregular margin and a nonparallel orientation is more likely to be seen as malignant, 2. due to limited data, why deep residual network performed worse than VGG16, could not be identified, 3.	Correct localization (CorLoc) measure 84.50%
49.	Almajalid et al. [53]	Breast lesion segmentation	U-Net	Two 3 × 3 convolution layers, 2 × 2 max pooling operation containing stride 2, batch size 8, epoch 300, learning rate 10^−5^	Minus dice	ReLU	1. Shortage of adequately labeled data, 2. kept only the largest false-positive regions, 3. failure case when no reasonable margin is detected.	Dice coefficient 82.52%
50.	Xiao et al. [69]	Breast masses discrimination	InceptionV3, ResNet50, and Xception, CNN3, traditional machine learning-based model	The input image sizes were 224 × 224, 299 × 299, and 299 × 299, respectively for ResNet50, Xception, and InceptionV3 models, Adam optimizer, batch size 16	Categorical cross-entropy	ReLU, softmax	1. When depth of fine-tuned convolutional blocks exceeds a certain target, overfitting occurs due to training a small-scale image samples, 2. Memory-consuming, cannot be applicable to embedded devices	Accuracy of Transferred InceptionV3, ResNet50, transferred Xception, and CNN3 were 85.13%, 84.94%,84.06%, 74.44%, and 70.55%,respectively
51.	Qi et al. [117]	Diagnosis of breast masses	Mt-Net, Sn-Net	Mini batch size 10, optimizer: ADADELTA, dropout 0.2, L2 regularization with λ of 10^−4^, input image size 299 × 299 pixels, used class activation map as additional inputs to form a region enhance mechanism, 1536 feature maps of 8 × 8 size in the Mt-Net, 2048 feature maps of 8 × 8 size in the Sn-Net	Cross-entropy	ReLU	Limitations not specified	Accuracy of Mt-Net BASIC, MIP AND REM are 93.52%, 93.89%, 94.48% and Sn-Net BASIC, MIP, and REM are 87.34%, 87.78%, 90.13%, respectively.
52.	Segni et al. [118]	Classification of breast lesions	S-detect	Not specified	Not specified	Not specified	1. Limited sample size, 2. High prevalence of malignancies, 3. Retrospective	Sensitivity > 90%, specificity 70.8%, ROC 0.81
53.	Zhou et al. [119]	Breast tumor classification	CNN	16 weight layers (13 convolution layers and 3 fully connected layers), 4 max-pooling layers, convolution kernel size was set 3 × 3, the numbers of convolution kernel for different blocks were 64, 128, 256, 512 and 512, the max-pooling size and strides were 2 × 2, Adam optimizer was used, batch size 8, maximal number of iterations 6400, initial learning rate 0.0001	Not specified	ReLU and Softmax	Not specified	Accuracy 95.8%, sensitivity 96.2%, and specificity 95.7%
54	Kumar et al. [54]	Breast mass segmentation	Multi U-Net	Dropout 0.6, optimizer: RMSprop, learning rate 5 × 10^−6^, convolution size 3 × 3 (stride 1), max-pooling size 2 × 2 (stride 2), input image size 208 × 208	Negative Dice coefficient	Leaky ReLU	1. The algorithm was trained using mostly BIRADS 4 lesions, limiting the model’s ability to learn the typical features of benign or malignant lesions, 2. limited training size, 3. Varying angle, precompression levels and orientation of the images limit the ability to better identify the boundaries of the masses. Different cross-sections’ information could not be combined.	Dice coefficient 84%
55.	Cho et al. [91]	To improve the specificity, PPV, and accuracy of breast US	S-Detect	Not specified	Not specified	Not specified	1. Small dataset, 2. Calcifications were not included in the study due to limited ability of the model to detect microcalcifications, nonmass lesions were also excluded, 3. Variation exists in selection of representative images, 4. 50.4% of the breast masses in this study were diagnosed by only core needle biopsy.	Specificity 90.8%, positive predictive value PPV 86.7%, accuracy 82.4, AUC 0.815
56.	Han et al. [120]	Classification of breast tumors	GoogLeNet	Momentum 0.9, weight decay 0.0002, a poly learning policy with base learning rate 0.0001, batch size is 32	Not specified	Not specified	1. More benign lesion than malignant ones, more sensitive to benign lesions, 2. ROIs should be manually selected by radiologists	Accuracy 0.9, sensitivity 0.86, specificity 0.96.
57.	Kim et al. [121]	Diagnosis of breast masses	S-detect	Not specified	Not specified	Not specified	1. US features analysis was based on the fourth edition of BI-RADS lexicon, changes in details may result in changes in results despite little has changed between 4th and 5th edition of BI-RADS, 2. No analysis of calcifications was performed with S-Detect, 3. Non-mass lesions were excluded, 4. One radiologist selected a ROI and a representative image, which could have differed if another radiologist was included.	Accuracy 70.8%
58.	Yap et al. [64]	Detection of breast lesions	A Patch-based LeNet, a U-Net, and a transfer learning approach with a pretrained FCN-AlexNet.	Iteration time t was 50, input patches are sized at 28 × 28, Patch based LeNet: Root Mean Square Propagation (RMSProp) was used, a learning rate of 0.01, 60 epochs, the dropout rate of 0.33, U-Net: Adam optimizer, a learning rate of 0.0001, 300 epochs, FCN-AlexNet: Stochastic gradient descent, a learning rate of 0.001, 60 epochs, a dropout rate of 33%	Patch-based LeNet: Multinomial logistic loss	ReLU and Softmax	They need a time-consuming training process and images that are normal.	Transfer Learning FCN-AlexNet performed best, True Positive Fraction 0.98 for dataset A, 0.92 for dataset B
59.	Antropova et al. [122]	Characterization of breast lesions	VGG19 model, deep residual networks	Automatic contour optimization based on the average radial, takes an image ROI as input, the model is composed of five blocks, each of which contains 2 or 4 convolutional layers, 4096 features were extracted from 5 max pooling layers, average pooled across the third channel dimension, and normalized with L2 norm, then the features which are normalized are concatenated to form the feature vector	Not specified	Not specified	1. The depth and complexity of deep learning layers for moderate sized dataset makes investigating their potential out of the scope of this experiment, 2. Single-center study	AUC = 0.90

**Table 3:** The descriptive comparative analysis across deep learning model performances among various stages of breast lesion management.

Purpose	Performance Metrics (No. of Studies)	Performance Mean ± Standard Error	Range	Maximum Achieved (Model)
**Segmentation**	Dice coefficient (9)	85.71 ± 1.55 (%)	79.62–96.95 (%)	96.96% (SegNet with the LNDF ACM)
	Accuracy (7)	94.69 ± 1.13 (%)	85–99.49 (%)	99.49% (LEDNet, ResNet-18, Optimal RNN, SEO)
**Classification**	Accuracy (20)	86.34 ± 1.69 (%)	50–100 (%)	100% (VGG16, ResNet34, and GoogLeNet)
	AUC (14)	0.87 ± 0.02	0.755–0.98	0.98 (VGG16 CNN)
**Prediction of ALN status**	Accuracy (8)	84.12 ± 2.50 (%)	74.9–98 (%)	98% (Feed forward, radial basis function, and Kohonen self-organizing)
	AUC (4)	0.88 ± 0.02	0.748–0.966	0.966 (Feed forward, radial basis function, and Kohonen self-organizing)
**Prediction of response to chemotherapy**	Accuracy (3)	87.9 ± 4.70 (%)	79.7–96 (%)	96% (ANN)
	AUC (2)	0.91 ± 0.05	0.86–0.96	0.96 (ANN)

## 8. Conclusions

Despite all these limitations, these deep learning models can save time and money in diagnosing a medical condition, which will reduce the workload of physicians so that they can spend quality time with patients. This has the potential to improve the quality of care and identify early management for the patients by automatically segmenting and classifying breast lesions into benign and malignant, or BI-RADS categories, to facilitate early management, monitoring response to chemotherapy, and progression of the disease, including lymph node metastasis with improved accuracy compared to radiologists and time efficiency. Moreover, in resource-limited areas, including low- and middle-income countries where breast cancer-related mortality is high due to a lack of physicians and radiology experts and, in some places, only ultrasound operators are making decisions, applying these deep learning models can considerably impact those scenarios [123,124,125]. The application of these models to real-world settings and the availability of these models and knowledge of deep learning to physicians are now a necessity.

## Data Availability

The data that support the findings of this study are available from the corresponding author upon reasonable request. The requested data may include figures that have associated raw data. Because the study was conducted on human volunteers, the release of patient data may be restricted by Mayo policy and needs special request. The request can be sent to: Karen A. Hartman, MSN, CHRC|Administrator—Research Compliance|Integrity and Compliance Office|Assistant Professor of Health Care Administration, Mayo Clinic College of Medicine & Science|507-538-5238|Administrative Assistant: 507-266-6286|hartman.karen@mayo.edu Mayo Clinic|200 First Street SW|Rochester, MN 55905|mayoclinic.org. We do not have publicly available Accession codes, unique identifiers, or web links.
